# Parkinsonism-Hyperpyrexia Syndrome: A Case Series and Literature Review

**DOI:** 10.7759/cureus.29646

**Published:** 2022-09-27

**Authors:** Jehad Azar, Yasmin Jaber, Mohammed Ayyad, Walaa Abu alia, Fahed Owda, Haneen Sharabati, Hiba Zeid, Suleiman Khreshi, Maram AlBandak, Dana Sayyed Ahmad

**Affiliations:** 1 Respiratory Institute, Cleveland Clinic, Cleveland, USA; 2 Oncology, Georgetown University Medical Center, Washington, D.C., USA; 3 Internal Medicine, Al-Quds University, Jerusalem, PSE; 4 Internal Medicine, Cleveland Clinic Fairview Hospital, Cleveland, USA; 5 Internal Medicine, An-Najah National University, Nablus, PSE; 6 Internal Medicine, Waterbury Hospital, Waterbury, USA; 7 Internal Medicine, St. Mary Medical Center, Langhorne, USA

**Keywords:** neuroleptic malignant syndrome, levodopa-carbidopa, dopaminergic withdrawal, deep brain stimulator, parkinsonism-hyperpyrexia syndrome, parkinson’s disease (pd)

## Abstract

Parkinsonism-hyperpyrexia syndrome (PHS) is a rare, potentially fatal neurological emergency, that is seen in Parkinson’s Disease (PD) patients and mimics neuroleptic malignant syndrome. The most common trigger for PHS is sudden withdrawal of anti-parkinsonian medications, specifically levodopa. However, it can also be due to Deep Brain Stimulation (DBS) device malfunction. In this work, we describe three cases of PHS; the first of which is related to DBS battery depletion, and the remaining two to dopaminergic withdrawal. Additionally, we will include the results of a literature review on PHS, its etiologies, presentation, and management.

## Introduction

Parkinson’s Disease (PD) is one of the most common progressive neurodegenerative disorders, affecting up to 3% of the population by age 65 [1]. The disorder is characterized clinically by cogwheel rigidity, rest tremor, bradykinesia, and asymmetry of symptoms. Pathologically, it is characterized by the loss of pigmented dopaminergic neurons in the substantia nigra pars compacta of the midbrain and the presence of Lewy bodies [2,3]. Thus, a definitive diagnosis of PD can only be obtained by autopsy, but skilled clinicians can characterize these pathological findings with fairly high accuracy. 

Although PD is not considered a life-threatening illness, it is nevertheless listed as the third-leading cause of death from a neurological condition (after stroke and Alzheimer's disease) and remains an incurable disease [4]. Therefore, management is aimed at improving quality of life (QoL) and alleviating symptoms, rather than halting or preventing disease progression. Physical therapy and exercise are likely beneficial to all patients and are often considered prior to initiating medical therapy, which is often an individualized decision [5].

Initial medical therapy is aimed at neuroprotection to maintain dopamine levels in dopaminergic neurons and provide symptomatic relief. Advanced management generally includes multiple drugs used in combination (e.g., carbidopa-levodopa along with entacapone, selegiline, amantadine, or dopamine agonists), and possibly surgical approaches. Treatment is tailored to the individual patient, taking into consideration factors such as age, gender, drug tolerance, and severity of symptoms [6].

Even with optimal medical therapy, patients may continue to experience debilitating symptoms, which has led to the development of interventional therapies. This includes surgical pallidotomy or thalamotomy, levodopa-carbidopa intestinal pumps, and high-frequency deep brain stimulation (DBS), achieved by implanting devices in targeted areas. The exact mechanism of action of high-frequency DBS remains an enigma even today. However, the clinical improvements seen in patients who receive DBS are so significant, they allow for a decrease in levodopa dosages, thereby markedly improving motor fluctuations, dyskinesias, and QoL [7-9]. The main drawbacks of DBS devices are the subsequent need for finetuning of settings, and battery replacement [10,11].

Parkinsonism-Hyperpyrexia Syndrome (PHS) is a life-threatening disorder, which manifests with pyrexia, muscular rigidity, autonomic instability, altered mental state (AMS), diaphoresis, and elevated creatinine phosphokinase (CPK) levels [12]. It is commonly reported following the withdrawal of anti-parkinsonian medications but has been observed following DBS device malfunction (i.e., battery depletion), before DBS surgery (when treatment is transiently withheld to observe the patient’s response in the “off” state), or when dosage reduction is attempted after the procedure. To the best of our knowledge, PHS following DBS battery depletion has been reported only in a few cases [13-15]. We herein present three cases of PHS, one of which is due to DBS battery depletion, and the other two to dopaminergic withdrawal, along with a comprehensive review of current literature. 

## Case presentation

Case one

A 76-year-old female patient presented to the emergency department with a four-day history of high-grade fever, poor oral intake, and altered mental status (AMS). Previous medical history was significant for ischemic cardiomyopathy, ischemic stroke, essential hypertension, hyperlipidemia, and type 2 diabetes mellitus (DM). She was diagnosed with PD at the age of 62, and was treated with levodopa-carbidopa, but due to poor control of her symptoms, underwent bilateral subthalamic nucleus (STN) DBS surgery five years after being diagnosed, which resulted in significant improvement. 

Upon admission she was febrile and tachycardic at 120 beats per minute (bpm); her blood pressure was 105/72 mmHg, and her respiratory rate was 22 breaths per minute. Oxygen saturation was 86% while breathing ambient air. Her physical examination showed a diaphoretic and severely dehydrated patient, in mild respiratory distress. A neurologic examination demonstrated somnolence with a lack of response to painful stimuli, severe muscular rigidity of all four extremities, and neck stiffness. Deep tendon reflexes of the knees, ankles, and biceps were 3+ (very brisk) bilaterally. Babinski’s sign was negative bilaterally, and there was no myoclonus. Her breath sounds were bilaterally diminished in the lung bases, with right lower lobe bronchial breathing and egophony. The remaining physical examination was unremarkable. 

**Figure 1:**
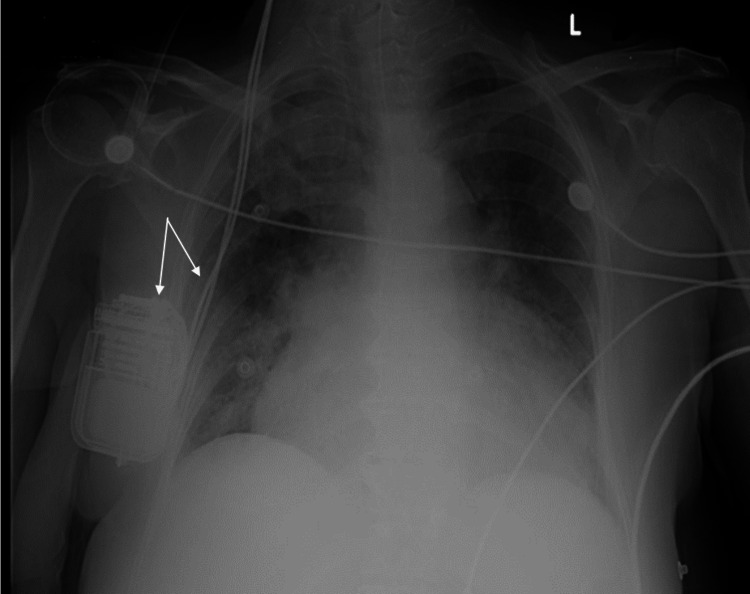
AP chest X-ray The image shows bilateral lower lobe infiltrates, more profound on the right, and cardiomegaly; the DBS device and leads are visible (white arrows). AP: Anteroposterior; DBS: Deep Brain Stimulation

Laboratory tests showed hypernatremia of 156 mmol/L, acute renal failure with a creatinine of 1.7 mg/dL, elevated CPK at 890 U/L, and leukocytosis 21,600 WBCs/µL (normal range 4,500-10,000 WBCs/µL), with a C-reactive protein (CRP) of 6.6 mg/dl (normal <0.3 mg/dl). A chest X-ray demonstrated bilateral infiltrates, more profound in the right lung, and cardiomegaly. The DBS device could be visualized over the right chest wall with percutaneous leads traveling through the proximal neck (Figure 1). 

Given the patient’s presentation and the above-mentioned findings, pulmonary infection was suspected. However, due to impaired consciousness and fever in an elderly patient, a lumbar puncture was completed to rule out CNS infection, with normal cell count and negative viral polymerase chain reaction (PCR) and CSF cultures. After blood, sputum, urinary, and stool cultures were obtained the patient was started on intravenous (IV) antibiotics and supportive therapy. However, after nearly a week, the patient’s symptoms did not resolve. She, therefore, underwent a whole-body CT, which failed to localize a possible source of infection and revealed no acute intracranial process. Respiratory culture and gram stains, respiratory viral panel, and other relevant cultures came back negative. The patient's thyroid function, calcium, ammonia, liver enzymes, and vitamin B12 levels were all within normal limits. 

She had no documented history of using a neuroleptic medication, and her dopaminergic medication was not altered (ruling out withdrawal), but given her known history of PD, PHS syndrome was suspected. As such, her levodopa dose was increased and she was started on IV fluids and acetaminophen. However, despite the correct diagnosis and appropriate management, her condition did not improve. At this point, PHS due to DBS battery depletion was suspected. Upon further investigation, it was discovered that her DBS battery had never been replaced, making the suspected diagnosis even more likely. 

**Figure 2:**
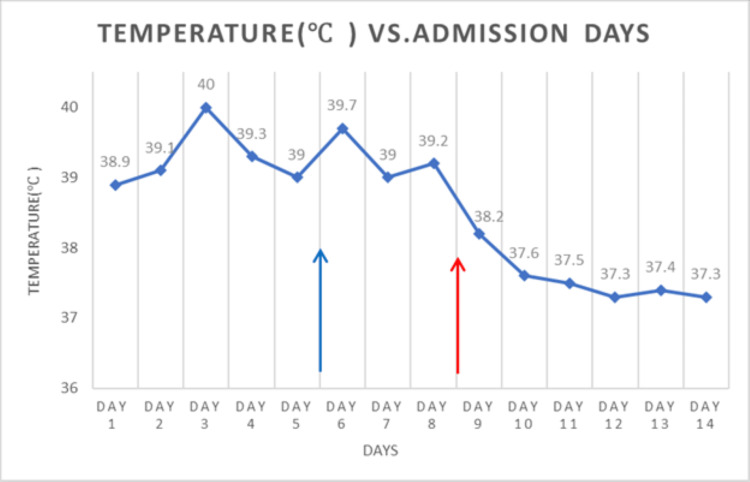
Fever chart of the patient Day 1 is the first day of admission. Day 6 (blue arrow) represents the time of increasing her Levodopa dose. Day 9 (red arrow) is when the IPG was replaced. On day 14 the patient was discharged to a rehabilitation facility. IPG: Implantable Pulse Generator

Successful Implantable Pulse Generator (IPG) replacement was performed on the ninth day of admission. Clinical improvement was documented a few hours later, and her fever and autonomic instability resolved the following day (Figure 2). Muscular rigidity and mental status gradually improved until recovery, and her leukocyte count, CPK, renal function, and CRP normalized.

Case two

A 73-year-old female patient presented to the emergency department with a four-day history of fever, productive cough, and shortness of breath. Her past medical history was significant for essential hypertension, major depression, type 2 diabetes mellitus, ischemic cardiomyopathy, and PD, diagnosed at the age of 64, and previously treated with levodopa-carbidopa. At age 67, with poor symptom control, a bilateral STN-DBS device was implanted, resulting in significant improvement of tremors and motor deficits.

Upon admission, she was febrile at 38.1°C, tachycardic at 105 bpm, and her respiratory rate was 32 breaths per minute with pulse oximetry of 86% breathing ambient air. On physical exam, she was oriented to person, place, and time. She showed signs of respiratory distress, with decreased air entry and crepitations over the right lower lung field. The remaining physical examination was unremarkable. Due to desaturation, she received oxygen supplementation by high-flow nasal cannula, with saturation improving to greater than 92%.

Laboratory tests were significant for leukocytosis at 15,600 WBCs/µL, with a CRP of 6.6 mg/dl. A chest X-ray demonstrated a right lower lobe infiltrate, suggesting pulmonary infection, and the patient was started on antibiotics for suspected aspiration pneumonia. A few hours after her admission, the patient deteriorated, with AMS, 39°C fever, and oliguria. A repeat physical exam showed significantly elevated blood pressure and tachycardia, and muscular rigidity. She became somnolent, diaphoretic, and was in respiratory distress. Repeated laboratory evaluation showed worsening leukocytosis of 17,000 WBCs/µL, acute kidney injury with a creatinine of 1.6 mg/dL, and blood urea nitrogen (BUN) of 32 mg/dL. CPK was elevated at 1400 U/L. She was managed for suspected sepsis due to pneumonia, with broad-spectrum antibiotics and IV fluids, yet only her respiratory symptoms improved. Fever, AMS, and muscular rigidity persisted, along with worsening autonomic instability. Subsequently, she underwent a broad inclusive evaluation that included blood, sputum, urinary, and stool cultures. She also underwent a lumbar puncture, ruling out CNS infection with a normal cell count and negative viral PCR and CSF cultures. Whole-body CT scan was performed, which failed to localize an alternate source of infection. Serology for cytomegalovirus (CMV) and Epstein-Barr virus (EBV), a respiratory viral swab, and all relevant cultures came back negative. The patient's thyroid functions, calcium, ammonia, liver enzymes, and vitamin B12 levels were all within normal limits. 

**Figure 3:**
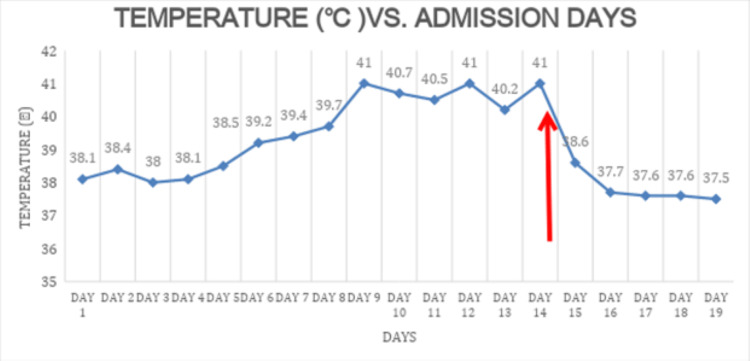
Fever chart Day 1 is the first day of admission. On day 15 (red arrow) Levodopa was resumed. On day 19 she was discharged to rehabilitation.

A thorough review of the patient’s history and previous medications was then completed, and while she had never been prescribed any neuroleptic agents in the past, it was discovered that her home levodopa-carbidopa was discontinued on admission and not resumed. As a result, the diagnosis of PHS due to dopaminergic withdrawal was made, 15 days after her initial admission. Subsequently, the patient was treated with IV fluids, acetaminophen, and ice packs; due to her somnolence, levodopa-carbidopa was administered through a nasogastric tube, with rapid clinical improvement, strongly supporting the diagnosis of PHS. Given the clinical improvement after resuming her dopaminergic agent, the DBS battery was not replaced. By admission day 18, her fever resolved (Figure 3). The patient's mental status, muscular rigidity, leukocytosis, and CPK level continued to improve gradually, until full recovery.

Case three

A 59-year-old female patient presented to the emergency department with a three-day history of fever, productive cough, shortness of breath, and AMS. Her previous medical history was significant for developmental delay, schizophrenia, recurrent urinary tract infections, recurrent aspirations, and PD. She had been diagnosed with PD at the age of 55, and treated with a combination of levodopa-carbidopa-entacapone, with adequate control of her symptoms. 

Upon admission, she was febrile at 38.4°C, tachycardic at 115 bpm; her respiratory rate was 28 breaths per minute, with pulse oximetry of 83% while breathing ambient air, which improved to 96% after receiving supplemental oxygen by nasal cannula. Her physical exam showed a patient in respiratory distress, with bilaterally decreased breath sounds, bronchial breathing, egophony over the right lower lobe, and coarse crepitations over the right lower lung. The patient’s neurological exam was positive for AMS; she was not oriented to time, place, or person. The remaining physical examination was unremarkable. 

Laboratory tests revealed leukocytosis of 14,500 WBCs/µL with a CRP of 12 mg/dl, and CPK of 265U/L. A chest X-ray revealed bilateral basal infiltrates, and a subsequent chest CT scan showed bilateral consolidations, and bilateral pleural effusion. She was started on antibiotics for suspected aspiration pneumonia. A few hours after her admission, the patient deteriorated, to the point of requiring sedation and endotracheal intubation. She was started on lung protective ventilation with volume control, 6-8 mL/kg, and high positive-end expiratory pressure (PEEP). Due to hypovolemia not being responsive to fluid resuscitation, she was started on IV norepinephrine. Propofol and morphine were used for deep sedation and analgesia.

Five days after her admission, given the improvement in her ventilatory parameters, she successfully completed a spontaneous breathing trial and was thereafter extubated, and received oxygen by nasal cannula at a rate of 3L/min. Despite her respiratory improvement, she continued to have a high fever of 39.5°C, worsening mental status, muscular rigidity, and diaphoresis, along with severely elevated blood pressure. Repeat laboratory evaluation showed worsening leukocytosis at 21,000 WBCs/µL, and a rising CPK (now 1700 U/L). Suspecting a non-resolving bacterial infection, her antibiotics were switched to a broad-spectrum regimen, yet over the next few days, she showed no improvement. A broad evaluation including blood, sputum, urinary, and stool cultures, as well as a lumbar puncture, was conducted, with no positive findings. She underwent an abdominal ultrasound to rule out abdominal pathology, as well as a duplex ultrasound to rule out deep vein thrombosis. A repeat whole-body CT scan was also negative for a source of infection. Serology for CMV and EBV, respiratory viral swab, and all relevant cultures came back negative. The patient's thyroid function, chemistry, ammonia, liver enzymes, and vitamin B12 levels were all within normal limits. The urinary toxicology screen was also negative. 

**Figure 4:**
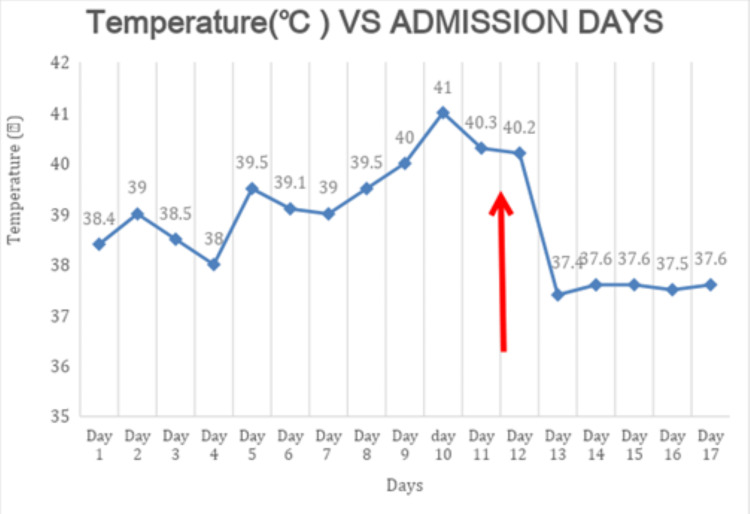
Fever chart Day 1 is the first day of admission. Day 12 (red arrow) represents the day of resuming carbidopa-levodopa-entacapone. On day 17 she was discharged to a rehabilitation facility

On day 12 of her admission, a thorough evaluation of the patient’s medication history was conducted, and while there was no documented use of neuroleptic medication, it was revealed that her home levodopa-carbidopa-entacapone medication was changed on admission to a levodopa-carbidopa agent at a lower maintenance dose. This crucial detail, combined with her current symptoms, led to the diagnosis of PHS due to dopaminergic withdrawal. Subsequently, her levodopa-carbidopa-entacapone was resumed at the correct pre-admission dose, and she was treated with IV fluids, acetaminophen, and ice packs with prompt clinical improvement. By the next day, her fever had resolved (Figure 4). Her remaining symptoms continued to improve gradually until full recovery, at which point she was discharged.

## Discussion

The differential diagnosis of PHS includes neuroleptic malignant syndrome (NMS) and serotonin syndrome. These conditions share features of dysautonomia, such as autonomic instability, pyrexia, and muscular rigidity. Hence, to distinguish between them, the treating physician must consider the setting in which they occurred and the implicated drugs. For instance, NMS develops minutes to hours after exposure to dopamine receptor blockers, whereas PHS mainly occurs in the setting of a dopamine agonist dosage reduction, withdrawal, or DBS malfunction. Serotonin syndrome may precipitate days after serotonergic drug usage, and may also present with additional clinical features such as myoclonus, hyperreflexia, seizures, and mood alterations. Other conditions to be considered in the differential diagnosis are malignant hyperthermia, malignant catatonia, and dyskinesia-hyperpyrexia syndrome [16,17].

Fever, AMS, and autonomic instability in an elderly patient may be the earliest manifestations of various pathological conditions. In the emergency room setting, elderly patients with multiple comorbidities often present with sepsis, a diagnosis with a mortality rate of approximately 26%, and requiring urgent interventions to improve outcomes [18]. PHS is another life-threatening medical emergency, yet because of the severe presentation in elderly patients, and the multi-organ damage incurred, it is not uncommon to misdiagnose PHS. As we have demonstrated, often patients will present with PHS in addition to a more frequently encountered condition (i.e., sepsis due to pneumonia), which can, unfortunately, confound the medical team and further delay accurate diagnosis, increasing mortality. In rare cases, patients may also present with an indolent infection of the DBS system, which requires expert consultation and individualized care [19]. 

The exact pathogenesis behind the development of PHS remains unclear. A growing body of evidence suggests that acute reduction of neurotransmission in the hypothalamus, the nigrostriatal system, and the mesocortical dopaminergic system contributes to the development of PHS [20]. It has been postulated that the underlying mechanism is similar to that of the neuroleptic malignant syndrome [21]. Complications of PHS include aspiration pneumonia, renal failure due to rhabdomyolysis, disseminated intravascular coagulation, and venous thromboembolism. In addition, patients may develop potentially fatal damage to the central nervous system (i.e. hypo-dopaminergic crisis) within hours to days. Supportive management and re-initiation of dopaminergic medications are the cornerstones of treatment [22]. 

In patients with DBS device implantation, abrupt withdrawal of DBS can induce PHS, regardless of changes in dopaminergic medications. The chronic high-frequency DBS may cause an adaptation phenomenon, causing a rebound effect, and rendering PHS due to DBS withdrawal unresponsive even to high doses of dopaminergic therapy. This also raises the possibility of different targets of action in the nigral pathway for oral therapy compared to DBS [13].

Physicians treating patients with long-term PD should always take note of their anti-parkinsonian regimen and strive to maintain it for the duration of their medical stay, with minimal change, if at all. In rare events where anti-parkinsonian medications need to be discontinued, even briefly, the decision should be made only after expert consultation. As we have shown, a thorough medical reconciliation, conducted in a timely manner, could have prevented the development of PHS altogether in our second and third cases.

Furthermore, physicians should have a low threshold for suspecting PHS in this specific population. This is especially true for patients who are chronically taking multiple dopaminergic medications, have long-standing PD, have had their DBS device placed for many years, or started showing signs consistent with PHS. 

Considering the benign nature of PHS treatment compared with the course of the syndrome itself, empirical treatment must be started promptly. This can be done by re-initiating dopaminergic medications with dosage increase when appropriate, along with supportive therapy of fluids, acetaminophen, and benzodiazepines as needed. Physicians should be especially aware that patients with DBS devices receiving dopaminergic therapy cannot be managed solely by battery replacement, and can suffer PHS if their medication is withdrawn, regardless of whether the DBS device is functioning or not. It is not rare for patients suffering from PHS to be somnolent or obtunded; in such cases, physicians may need to resort to alternative means to administer medications, such as by using a nasogastric tube, as was the case with our first patient. As demonstrated in the literature, rapid and drastic response to treatment is highly supportive of PHS diagnosis. Additional triggers of PHS reported in the PD patient include prescription of neuroleptic medication, acute infection, dehydration, and exposure to extreme heat. 

Literature review

Cases of PHS reportedly associated with acute DBS withdrawal are summarized in Table 1. 

**Table 1:** Reported cases with DBS withdrawal syndrome, in comparison with the patients presented in this work S: sex; DBS: Deep brain stimulation; PDD: Parkinson's disease duration; DBSD: DBS duration at PHS onset; CPK: Creatinine phosphokinase; CRP; C-reactive protein; IPG: Implantable pulse generator Conservative treatment refers to intravenous (IV) fluids, antipyretics, antibiotics, cooling measures, and sedatives.

Report	Age (y)/S	PDD/DBSD	Treatment	Lab results	Cause of DBS failure	Treatment	Outcome
Artusi et al. [13]	63/M	18/5	↑Levodopa, conservative	↑CPK (2820 U/L) ↑CRP (50.1 mg/L) ↑WBC (10,000/μL	Battery depletion	IPG was re-implanted	Recovery
Neuneier et al. [14]	77/M	18/5	↑Levodopa, Amantadine, conservative	↑CPK (1642 U/L) ↑CRP (50 mg/L)	Battery depletion	Late IPG re-implantation	Death
Liu et al. [15]	69/M	16/9	↑Levodopa, Bromocriptine, Dantrolene, conservative	↑CPK (1250 IU/L) ↑WBC (12,100/μL)	Battery depletion	Battery was replaced	Recovery
Hocker et al. [17]	74/M	-/4	Carbidopa-Levodopa, Dantrolene, conservative	↑CPK (2845 U/L)	DBS switched off	DBS switched on & IPG replaced	Recovery
Reuter et al. [23]	75/M	19/9	↑Levodopa, Amantadine, conservative	-------	IPG infection	IPG wasn’t re-implanted	Death
Reuter et al. [23]	74/M	24/10	↑Levodopa, Amantadine, conservative	-------	IPG infection	IPG wasn’t re-implanted	Death
Reuter et al. [23]	52/M	20/8	↑Levodopa, Amantadine. Apomorphine, conservative	-------	IPG infection	IPG was re-implanted	Recovery
Kadowaki et al. [24]	60/M	17/8	Conservative	↑CPK (1878 U/L) ↑CRP (10.3 mg/L) ↑WBC (12,600/μL)	DBS switched off	DBS switched on	Recovery
Rajan et al. [25]	51/M	11/7	Levodopa/Carbidopa, Amantadine, Domperidone, conservative	↑CPK (1878 U/L)	Battery depletion	IPG replacement	Recovery
Azar et al. [26]	67/F	23/7	↑Levodopa, Pramipexole, conservative	↑ CPK (1615 U/L) ↑ CRP (10.6 mg/L) ↑WBC (16.500/μL)	Battery depletion	Battery replacement	Recovery
Han et al. [27]	69/F	24/-	Levodopa/benserazide, Trastal, Amantadine, conservative	↑WBC (18.0 × 109/L)	Medication withdrawal after DBS surgery	anti-parkinsonian medication resumed	Recovery
Kim et al. [28]	66/F	11/-	Levodopa, Dopamine Agonists, conservative	↑CPK (786 U/L)	Medication withdrawal after DBS surgery	anti-parkinsonian medication resumed	Recovery
Urasaki et al. [29]	75/F	14/-	↑Levodopa/benserazide, conservative	↑CPK (3711 U/L) Positive cardiac troponin	Medication withdrawal after DBS surgery	anti-parkinsonian medication dosage increased	Death
Akçakaya et al. [30]	61/M	14/-	Levodopa/benserazide, Trihexyphenidyl, Amantadine, Bromocriptine, IV Fluids, Vancomycin	↑CRP (3431) mg/L ↑CPK (2170 U/L)	Medication withdrawal after DBS surgery	anti-parkinsonian medications resumed and DBS system activated	Recovery
Our first case	76/F	14/9	↑Levodopa, conservative	↑ CPK (1250 U/L) ↑ CRP (6.6 mg/L) ↑WBC (21.500/μL)	Battery depletion	Battery replacement	Recovery
Our second case	73/F	9/5	Levodopa/carbidopa	↑ CPK (4,380 U/L) ↑ CRP (6.6 mg/L) ↑WBC (1700/μL)	Dopaminergic agent withdrawal	anti-parkinsonian medication resumed	Recovery
Our third case	59/F	-------	Levodopa/carbidopa/entacapone	↑ CPK (1700 U/L) ↑WBC (21 000/μL)	Dopaminergic agent withdrawal	anti-parkinsonian medication resumed	Recovery

Artusi et al. described a 63-year-old male with long-standing advanced PD with suspected PHS due to DBS battery depletion, showing gradual clinical and laboratory improvement after IPG replacement [13]. Neuneier et al. reported a case of fatal PHS in a patient with advanced PD and coronary heart disease treated with aspirin, who developed withdrawal syndrome a few days after battery depletion [14]. IPG replacement was postponed in this case, due to the elevated risk of bleeding, and his dopaminergic medication dose was sharply increased. His condition worsened, however, resulting in the death of the patient with disseminated intravascular coagulation (DIC) and multiorgan failure. This case also highlights the adaptation phenomenon seen in PD patients treated with DBS, their poor response to oral dopaminergic therapy, and the importance of early surgical intervention in such cases. Reuter et al. published three cases with PHS, after the removal of a DBS implant, due to hardware-related infection [23]. Fatal outcomes were reported in patients who had no IPG replacement despite an increase in the dose of levodopa. Liu et al. reported a patient with a history of PD for 16 years, who developed PHS during preoperative assessment for planned DBS battery replacement, which was consequently postponed on account of suspected sepsis [15]. Following significant clinical deterioration despite broad-spectrum antibiotics administration, and the failure to identify a source of sepsis, PHS was suspected. The patient was treated with dantrolene and bromocriptine, increasing the dose of dopaminergic medications, as well as intense supportive care. As a result of the failure of conservative management, the DBS battery was replaced with subsequent recovery. Kadowaki et al. described a PD patient with minor depression, who developed prominent manic symptoms following STN-DBS [24]. After switching off the DBS devices, his manic symptoms disappeared, but he developed recurrent PHS with each attempt. Ultimately, during one manic episode, low voltage stimulation was applied, preventing the development of PHS and eliminating the manic symptoms. 

Rajan et al. described a 51-year-old male patient who developed PHS seven years after DBS device implantation [25]. Intensive medical and conservative therapy were initiated but the patient showed no response to treatment. On day 11 post-admission the IPG was replaced, which led to marked improvement of the patient’s signs, symptoms, and laboratory findings. He was then discharged on medical therapy and returned to his baseline state of health and activities. Hocker et al. described a 74-year-old male who was admitted for dizziness and falls [17]. The patient’s physical examination on admission was significant for diffuse rigidity but no other signs. The next day, the patient developed signs and symptoms consistent with PHS and was treated with anti-parkinsonian medication, dantrolene, and conservative therapy. Due to the suspicion of PHS, the DBS device was checked and was found to be off. It was then reactivated and resulted in rapid improvement of the patient’s signs and symptoms. Due to the unexpected malfunction in the device, the IPG unit was replaced, and the patient was discharged from the nursing home.

Azar et al. described a 67-year-old female patient who presented to the emergency room with fevers, AMS, and poor oral intake [26]. Medical history was significant for PD diagnosed at the age of 44, treated with anti-parkinsonian medication and subsequent DBS implantation due to disease progression and inadequate control. Physical exam on admission demonstrated autonomic instability and muscle rigidity, and signs of a pulmonary infection. Laboratory findings showed elevated creatine kinase (CK), CRP, and leukocytosis. The patient was treated for suspected pneumonia but showed no improvement; imaging and further investigations didn’t reveal an infectious etiology or otherwise, which led to suspicion of PHS. The patient was started on conservative therapy along with increasing the dosages of her dopaminergic medications, with no marked response. DBS battery depletion was suspected, and the patient then underwent IPG replacement. After the procedure was performed, the patient’s signs, symptoms, and laboratory findings improved significantly within hours and she was discharged to a rehabilitation center.

Regarding PHS in the perioperative period, Han et al. reported a 69-year-old female patient who developed worsening PD symptoms despite being on medical therapy [27]. The patient was a good candidate for STN-DBS and underwent surgery after her PD medications were withheld preoperatively. Shortly after surgery, the patient developed PHS. Her PD medications were resumed and her symptoms and vital signs rapidly improved. Kim et al. reported a 66-year-old female patient with PD, who was admitted to the hospital for DBS surgery due to persistent symptoms despite optimal medical treatment, and severe side effects [28]. Anti-parkinsonian medications were withheld two days preoperatively and the surgery was uneventful. However, the patient developed PHS a few hours postoperatively. Anti-parkinsonian medications were administered along with conservative therapy and the patient showed rapid and drastic improvement in her signs and symptoms. Urasaki et al. described a 75-year-old female patient with a 14-year history of gradually progressive PD despite treatment [29]. DBS surgery was scheduled and three out of six of the patient’s antiparkinsonian medications were stopped prior to surgery. Days after surgery, the patient developed dyskinesia and was resolved by increasing the dose of amantadine. She then developed signs and symptoms consistent with PHS and was treated with IV fluids and levodopa/benserazide, which led to an improvement in her vital signs. However, despite treatment, the patient was agitated and had severe tremors, hallucinations, and bradykinesia. Her fever recurred and she suffered fatal cardiac arrest. Laboratory findings showed elevated CPK and cardiac troponins - indicating progression of PHS accompanied by a myocardial infarction. Akçakaya et al. described a 61-year-old male patient with a 14-year history of PD admitted for DBS surgery [30]. The procedure was uneventful. However, six days postoperatively the patient suffered from fever, tremor, rigidity, and autonomic instability. IV fluids and empiric antibiotics were started but showed no response. Laboratory findings showed elevated CPK which led to suspicion of PHS. In addition to bromocriptine, his usual anti-parkinsonian medications were restarted, but the patient still showed no improvement. A decision to activate the DBS device was made, and bromocriptine dose was increased. This led to a significant improvement in the patient’s signs, symptoms, and laboratory values. The patient then fully recovered and was discharged home.

In our first reported case, we highlight the importance of routine DBS device maintenance and battery replacement, which is potentially lifesaving, as well as the exclusion of other possible etiologies, which could confound the diagnosis. PHS was suspected despite a scarcity of previously reported cases. Initial treatment involved the increase of levodopa dose, administration of IV fluids, as well as pramipexole, with no clinical improvement in a patient with advanced PD and long-term STN stimulation. It was only after IPG replacement that the patient began to show signs of recovery.

In the second case, a PD patient with a DBS implant presented to the hospital with sepsis due to pneumonia and was managed accordingly. However, a few hours after admission, the patient’s condition deteriorated. Following an extensive workup, the patient was diagnosed with PHS due to the inappropriate discontinuation of her dopaminergic medications upon admission. Her rapid recovery after resuming dopaminergic medication shows the potentially life-threatening complication of dopaminergic withdrawal, even in patients with a functioning DBS device. Along with our first case, these two cases highlight the importance of carefully assessing the patient as a whole and familiarizing oneself with common disorders such as PD, their management, and possible complications. 

Finally, in the third case, a patient with multiple comorbidities presented with severe pneumonia and respiratory failure requiring endotracheal intubation, which was managed appropriately. However, her medication was erroneously changed abruptly, which led to the severe manifestations of PHS mentioned above. In both this and our second case, it was the medical team’s diligence and thorough evaluation which led to the breakthrough discovery and subsequent diagnosis. 

As can be inferred from Table 1, possible risk factors for life-threatening DBS withdrawal syndrome may include: long-standing PD (mean 19.3 years), prolonged DBS stimulation (mean 7.6 years), and old age (mean 67.1 years). An optimal prognosis can be achieved with a high index of suspicion and immediate DBS restoration, while delayed restoration or failure to restore DBS activity can result in fatal outcomes.

## Conclusions

PHS is a rare, life-threatening condition occurring in PD patients. The cases we have presented herein, and existing literature, demonstrate how easily a patient with PHS can be misdiagnosed. Hence, for any PD patient with acute deterioration of unknown etiology, one must have a high index of suspicion for PHS. A careful clinical history, drug review, and an inquiry on the DBS device leads to the correct diagnosis in most cases. Treatment must be individualized but includes supportive therapy and dopaminergic medications with appropriate dosage modification. In all cases of PHS, expert consultation is recommended and should be initiated without delay. 

## References

[1] Cerri S, Mus L, Blandini F (2019). Parkinson's disease in women and men: what's the difference?. J Parkinsons Dis.

[2] Dauer W, Przedborski S (2003). Parkinson's disease: mechanisms and models. Neuron.

[3] Reich SG, Savitt JM (2019). Parkinson's disease. Med Clin North Am.

[4] Sethi S, Hohler AD (2016). The application of palliative care principles in advanced Parkinson’s disease. Advances in Parkinson's Disease.

[5] Fox SH, Katzenschlager R, Lim SY (2018). International Parkinson and movement disorder society evidence-based medicine review: Update on treatments for the motor symptoms of Parkinson's disease. Mov Disord.

[6] Raza C, Anjum R, Shakeel NU (2019). Parkinson's disease: mechanisms, translational models and management strategies. Life Sci.

[7] Benabid AL, Chabardes S, Mitrofanis J, Pollak P (2009). Deep brain stimulation of the subthalamic nucleus for the treatment of Parkinson's disease. Lancet Neurol.

[8] Kleiner-Fisman G, Herzog J, Fisman DN (2006). Subthalamic nucleus deep brain stimulation: summary and meta-analysis of outcomes. Mov Disord.

[9] Moro E, Lozano AM, Pollak P (2010). Long-term results of a multicenter study on subthalamic and pallidal stimulation in Parkinson's disease. Mov Disord.

[10] Bronstein JM, Tagliati M, Alterman RL (2011). Deep brain stimulation for Parkinson disease: an expert consensus and review of key issues. Arch Neurol.

[11] Bloem BR, Okun MS, Klein C (2021). Parkinson's disease. Lancet.

[12] Newman EJ, Grosset DG, Kennedy PG (2009). The parkinsonism-hyperpyrexia syndrome. Neurocrit Care.

[13] Artusi CA, Merola A, Espay AJ, Zibetti M, Romagnolo A, Lanotte M, Lopiano L (2015). Parkinsonism-hyperpyrexia syndrome and deep brain stimulation. J Neurol.

[14] Neuneier J, Barbe MT, Dohmen C, Maarouf M, Wirths J, Fink GR, Timmermann L (2013). Malignant deep brain stimulation-withdrawal syndrome in a patient with Parkinson's disease. Mov Disord.

[15] Liu CJ, Crnkovic A, Dalfino J, Singh LY (2017). Whether to proceed with deep brain stimulator battery change in a patient with signs of potential sepsis and Parkinson hyperpyrexia syndrome: a case report. A A Case Rep.

[16] Grover S, Sathpathy A, Reddy SC, Mehta S, Sharma N (2018). Parkinsonism-hyperpyrexia syndrome: a case report and review of literature. Indian J Psychiatry.

[17] Hocker S, Kenney DL, Ramar K (2013). Parkinsonism-hyperpyrexia syndrome: broadening our differential diagnosis in the ICU. Neurol Clin Pract.

[18] Purcarea A, Sovaila S (2020). Sepsis, a 2020 review for the internist. Rom J Intern Med.

[19] Kim MS, Jeong JS, Ryu HS, Choi SH, Chung SJ (2017). Infection related to deep brain stimulation in patients with Parkinson disease: clinical characteristics and risk factors. J Neurol Sci.

[20] Toru M, Matsuda O, Makiguchi K, Sugano K (1981). Neuroleptic malignant syndrome-like state following a withdrawal of antiparkinsonian drugs. J Nerv Ment Dis.

[21] Granner MA, Wooten GF (1991). Neuroleptic malignant syndrome or parkinsonism hyperpyrexia syndrome. Semin Neurol.

[22] Mizuno Y, Takubo H, Mizuta E, Kuno S (2003). Malignant syndrome in Parkinson's disease: concept and review of the literature. Parkinsonism Relat Disord.

[23] Reuter S, Deuschl G, Falk D, Mehdorn M, Witt K (2015). Uncoupling of dopaminergic and subthalamic stimulation: life-threatening DBS withdrawal syndrome. Mov Disord.

[24] Kadowaki T, Hashimoto K, Suzuki K, Watanabe Y, Hirata K (2011). Case report: recurrent parkinsonism-hyperpyrexia syndrome following discontinuation of subthalamic deep brain stimulation. Mov Disord.

[25] Rajan R, Krishnan S, Kesavapisharady KK, Kishore A (2016). Malignant subthalamic nucleus-deep brain stimulation withdrawal syndrome in Parkinson's disease. Mov Disord Clin Pract.

[26] Azar J, Elinav H, Safadi R, Soliman M (2019). Malignant deep brain stimulator withdrawal syndrome. BMJ Case Rep.

[27] Han CL, Ge Y, Meng DW, Zhang JG, Meng FG (2017). Parkinsonism-hyperpyrexia syndrome after withdrawal of antiparkinsonian drugs and deep brain stimulation surgery. Chin Neurosurg J.

[28] Kim JH, Kwon TH, Koh SB, Park JY (2010). Parkinsonism-hyperpyrexia syndrome after deep brain stimulation surgery: case report. Neurosurgery.

[29] Urasaki E, Fukudome T, Hirose M, Nakane S, Matsuo H, Yamakawa Y (2013). Neuroleptic malignant syndrome (parkinsonism-hyperpyrexia syndrome) after deep brain stimulation of the subthalamic nucleus. J Clin Neurosci.

[30] Akçakaya MO, Akçakaya NH, Kasımcan MÖ, Kırış T (2018). Life-threatening parkinsonism-hyperpyrexia syndrome following bilateral deep brain stimulation of the subthalamic nucleus. Neurol Neurochir Pol.

